# Age–related changes in the metabolism and stress biomarkers of breeder hens

**DOI:** 10.1016/j.psj.2026.107949

**Published:** 2026-10-14

**Authors:** Raúl Caballero-Falcó, Antonio González de Bulnes, Paula Martínez-Ros, Juan Carlos Abad, Cristina Guerra, M. Carmen López-Mendoza

**Affiliations:** a Department of Animal Production and Health, Public Veterinary Health and Food Science and Technology, Universidad Cardenal Herrera-CEU, CEU Universities, Valencia, Spain; b Technical service, Cobb Española S.A, Palencia, Spain

**Keywords:** Breeder-hen, Genetics, Life cycle, Metabolism, Stress

## Abstract

Our study aimed to evaluate reference intervals, interrelationships and changes over the lifespan of different metabolic and stress biomarkers of two different genetic lines of breeder (grandparent) hens (one female line, *n* = 290 and one male line, *n* = 290), either in blood or feather. Serum metabolic biomarkers included total proteins, albumin, uric acid, total cholesterol, high- and low-density lipoproteins (HDL-c and LDL-c), triglycerides, glucose, and non-esterified fatty acids. Stress biomarkers included feather corticosterone and the heterophil-to-lymphocyte (H:L) ratio in blood. Age markedly influenced all biomarkers, specifically with significant and progressive increases in lipids and proteins related to aging and more marked fluctuations in glucose and non-esterified fatty acids (NEFA). Feather corticosterone also increased progressively over time, whilst no significant age-related differences were detected for the H:L ratio. Significant differences were observed between pre-laying and post-laying periods for most biomarkers, except for NEFA and H:L ratio, reflecting the increased metabolic demands associated with the onset and maintenance of egg production. Correlation analyses revealed significant associations between metabolic and stress biomarkers, suggesting an interaction between metabolic status and stress response. Overall, these findings improve the understanding of age-related changes in metabolism and stress physiology in breeder hens and provide reference values that may support health monitoring, nutritional adequacy, welfare assessment, and the development of more sustainable poultry production systems.

## Introduction

Breeder hens, being the primary source of genetic material and progeny for commercial production, are underpinning the entire poultry production pyramid. In such system, their performance is continuously challenged by their proper intensive production cycle, which causes high metabolic and endocrine demands that determine their productive yields; follicular development, ovulation, and egg formation require substantial energy and nutrient supply (Assersohn et al., 2021). Even before egg laying starts the birds go through an intensive phase of body growth and reproductive maturation, specially marked by the development of the oviduct and follicular activation (Ma et al., 2025). The beginning of laying, from 20 to 26 weeks-old, and the production peak, when egg quantity and quality are highest at around 26–33 weeks-old, are highly dependent on adequate management, nutrition and health/welfare (Tůmová et al., 2017; Kazemi et al., 2022). Afterwards, egg production enters a period of slow, progressive age-related decline (Arrazola et al., 2022), egg weight and quality may have slight variations depending on genetics and breeding conditions (de Oliveira et al., 2026) . From 48 weeks-old onwards, the hen enters its final laying phase (Cobb-Vantress, 2020), characterized by a marked decline in quantity and quality, influenced by physiological aging, which can be moderated by nutritional and environmental adjustments (Wang et al., 2018). Consequently, body composition, metabolic state and health/welfare status play a key role in sustaining egg production over time (van Eck et al., 2023).

Issues related to health and welfare are thus common in intensive production and any imbalance may even compromise the appropriate function of the breeder by challenging fertility and reproductive performance and progeny quality (Zuidhof et al., 2015; van Eck et al., 2023; Cash et al., 2025). Accordingly, surveillance and maintenance of health, welfare and performance of breeder flocks are aspects of paramount importance. Hence, in view of their usefulness for anticipating health and production problems (Alagawany et al., 2014; Kareem et al., 2024). The interest on assessment of health and welfare biomarkers in hens has increased in recent years. However, conversely to broiler lines (Martin et al., 2010; Awad et al., 2020; Negari et al., 2024), there is little information on breeder hens and, currently, there are no consolidated reference values for biochemical profile or main stress/disease biomarkers throughout the life cycle. Therefore, establishing specific reference values for physiological and stress biomarkers is of great interest, as they provide early information on the birds’ health status, adaptation to environmental and management conditions, and potential deviations that could affect reproductive performance and chick quality (Livingston et al., 2020; Nwaigwe et al., 2020; Arzour-Lakehal and Boudebza, 2021; Mohamed M. et al., 2022).

Assessment of health status and nutritional and physiological conditions, as in other species, is routinely based on the analysis of different blood biochemical parameters (Zálešáková et al., 2025). Markers related to protein metabolism and renal function (total protein, albumin, and uric acid), lipid metabolism and energy balance (total cholesterol, low-density lipoprotein [LDL-c], high-density lipoprotein [HDL-c], triglycerides and non-esterified fatty acids [NEFA]), and carbohydrate metabolism (glucose) provide integrated information on metabolism and energy status, and therefore the birds’ capacity to meet the physiological demands of egg laying. On the other hand, stress in birds is manifested through endocrine, immunological, and behavioral responses. The sympathetic-adrenomedullary and hypothalamic-pituitary-adrenocortical axes are the primary mediators of the rapid response to stressors, releasing catecholamines by brainstem neurons and glucocorticoids from the adrenal glands (Smulders, 2021). In birds, corticosterone is the main glucocorticoid while cortisol is produced only in very low amounts (Scanes, 2016). These glucocorticoids also have a clear impact on the blood profile, increasing heterophils and decreasing lymphocytes, which allows the heterophil:lymphocyte ratio (H:L) to be used as a reliable stress indicator (Mehaisen et al., 2017). The integration of endocrine and hematological responses provides a more comprehensive understanding of the birds' adaptation to their environment and physiological changes throughout their life cycle.

However, as previously explained and despite the relevance of these aspects, there is a gap in the literature regarding the assessment of physiological values and changes over time on metabolic and stress biomarkers in breeder hens. The availability of such specific reference values would allow the establishment of more accurate standards for monitoring animal health and welfare. Hence, the present study aimed to establish indicative reference intervals of selected metabolic and stress-related biomarkers and characterize their lifetime evolution in breeder hens, either in blood or in non-invasive samples (feathers).

## Materials and methods

### Bird husbandry

The study involved a total of 580 breeder hens (*Gallus gallus domesticus*) from two Cobb grandparent lines; a female line (FL; *n* = 290) and a male line (ML; *n* = 290). Four independent barns, environmentally controlled with dynamic and tunnel ventilation complemented by pad-cooling during summer, and equipped with the same temperature regulation systems, were used in the same commercial farm (Cobb Española SA, Dueñas, Palencia, Spain; April 2024 to June 2025): one rearing barn and one production barn for each of the FL and ML lines. Hens of both lines were reared separately and transferred to the production barn at 21 weeks of age. Nests were automatic communal nests with perches 1.8 m long on each side. Stocking density was 5.38 birds/m² in the FL barn and 4.06 birds/m² in the ML barn, and the male:female ratio was 1:10. Birds were fed daily under a restricted feeding program, the feeder space was 17.5 cm per animal for the ML line and 16.3 cm per animal for the FL line; the amount of feed provided per ration (g/bird/day) is shown in Table 1. Body weight and egg production relative to the Cobb breed standard for both lines are shown in Supplementary Figures S1 and S2, respectively. Thus, both lines were managed under similar husbandry conditions. In the ML house, ambient temperature averaged 25.82 ± 3.71 °C during the rearing phase and 22.47 ± 1.34 °C during the production phase. In the FL house, temperatures averaged 26.13 ± 3.91 °C during rearing and 22.43 ± 1.51 °C during production. The lighting cycle was adjusted according to the age of the birds: 23 h of light during the first week; 18 h during weeks 1–2; 12 h during weeks 2–3; 8 h from weeks 3 to 21; 12 h during weeks 21–22; 13 h during weeks 22–23; and 14 h from week 23 until the end of the life cycle. The composition of the diet is shown in Table 2.

**Table 1:** Feeding program for both breeder hens genetic lines across age.

Male Line (ML)		Female Line (FL)	
Age (weeks)	g/bird/day	Age (weeks)	g/bird/day
1	40	1	27
4	63	4	43
8	65	8	53
12	72	12	63
16	74	16	80
20	85	20	77
26	121	26	140
31	122	31	165
48	117	48	163
60	117	60	163

**Table 2:** Nutrient composition of the diets fed to breeder hens across age.

Age	kcal	%PB	%Lys DA
1–4 weeks	2940	19–60	0–960
5–15 weeks	2700	14–52	0–600
16–24 weeks	2800	15–00	0–635
25–45 weeks	2800	15–00	0–663
46–60 weeks	2800	14–50	0–636

PB, crude protein; Lys DA, digestible lysine; kcal, kilocalories per kg of feed.

### Blood and feather collection

Twenty hens per genetic line were individually sampled at the first week of life; afterwards, 30 hens per genetic line were individually sampled at key time points throughout the life cycle (1, 4, 8, 12, 16, 20, 26, 31, 48, and 60 weeks-old). Blood and feather samples were collected from each animal in the early morning, before access to feed, and according to the European Union Directive and the Spanish Policy for Animal Protection RD53/2013. The Committee of Ethics in Animal Research of Universidad CEU-Cardenal Herrera approved the experiment (report CEEA 26/30).

At each sampling time, blood samples (2 mL) were drawn from the brachial vein. A portion of 1 mL was transferred into EDTA tubes for determination of the heterophil-to-lymphocyte (H:L) ratio immediately after recovery, while the remaining blood sample (around 1 mL) was collected into plain tubes to obtain the serum for biochemical analyses. After allowing clotting for 2 h at room temperature, samples were centrifuged to separate serum, which was stored at −20 °C until analyzed. The H:L ratio was not determined at the first week of age, since the small body size of the chickens may compromise their welfare.

Concomitantly to blood sampling, two primary wing feathers were obtained through manual plucking from 4 weeks of age; sampling was not performed at one week of age because of the small body size of the chickens. Feathers were placed in sealed plastic bags, transported to the laboratory under refrigerated conditions and stored at −20 °C until analysis. The same feather type and position were sampled across all ages to ensure consistency, and any damaged or contaminated feathers were excluded.

### Assessment of metabolic biomarkers

The following biochemical parameters were determined in serum samples: total protein, albumin, uric acid, total cholesterol, LDL, HDL, triglycerides, glucose and NEFA. The analyses were performed using a Konelab 20 automated clinical biochemistry analyzer (Thermo Fisher Scientific, Vantaa, Finlandia) and commercial reagents according to the manufacturers’ instructions. Total protein, albumin, uric acid, total cholesterol, LDL, HDL, triglycerides, and glucose were determined using reagents from Spinreact SAU (Sant Esteve de Bas, Girona, Spain), including Total protein (Ref. 1001291), Albumin (Ref. 1001020), Uric acid (Ref. 1001010), Cholesterol-LQ (Ref. 41022), LDL cholesterol D (Ref. SP41023), HDL cholesterol D (Ref. 1001098), Triglycerides (Ref. 1001311), and Glucose-TR (Ref. 1001190). Non-esterified fatty acids were measured using the NEFA-HR (2) assay from FUJIFILM Wako Chemicals Europe GmbH (Neuss, Germany), including the R1 set (Ref. 434-91795) and R2 set (Ref. 436-91995). All analytical methods used had been previously applied in poultry species. Spinreact and FUJIFILM reagents and automated clinical chemistry analyzers, as used in the present study, have been previously employed to determine serum biochemical parameters in chickens and laying hens (Viveros et al., 2007; Marono et al., 2017; Spears et al., 2024; Katsumata et al., 2024; Semida et al., 2025). Internal quality controls were included in each series of analyses to verify the precision and accuracy of the results.

### Assessment of corticosterone in feather

Feather corticosterone was extracted following the procedure described by Bortolotti et al. (2008), with minor modifications previously validated by Häffelin et al. (2020). Thus, feathers were washed with distilled water after thawing and allowed to completely air‑dry at room temperature. The barbs were then separated from the rachis using a scalpel, cut into fragments smaller than 5 mm² and weighed with an analytical balance (AS 220.R1 PLUS, Radwag, Radom, Poland; maximum capacity 220 g, accuracy 0.1 mg) for having a 10 mg sample. Such samples were then transferred into disposable 50‑mL screw‑cap centrifuge tubes (Fisherbrand, cat. no. 05‑539‑9; Thermo Fisher Scientific, Waltham, MA, USA) and centrifuged (4000 rpm, 1 min) for ensuring complete recovery of the material and preventing sample loss due to adherence to the tube walls. Immediately, 10 mL HPLC‑grade methanol (Fisher Scientific, Waltham, MA, USA) were added to each tube and samples were sonicated in a room‑temperature water bath for 30 min. Samples were subsequently incubated at 50°C in a thermomixer (Thermo Shaker PST‑60HL‑4, Biosan, Riga, Latvia) at 189 rpm for 12 h. After incubation, the methanol extract was recovered by filtering the feather residues through syringe filters (25‑mm, 0.22‑µm; Scharlau Syringe Filters, Scharlab S.L., Sentmenat, Barcelona, Spain). Feather residues were washed twice with 1 mL of methanol each time, and the washings were combined with the original extract. The collected methanol was evaporated in a 50 °C water bath until completely dried. The dry extracts were then resuspended in 500 µL of assay buffer (tris‑buffered saline) supplied with the ELISA kit (Assay Buffer 15 by Enzo Life Sciences Inc., New York, NY, USA). Corticosterone quantification was performed using the Enzo Life Sciences Corticosterone ELISA Kit ADI-900-097 (Enzo Life Sciences Inc., New York, NY, USA), following the manufacturer's instructions. Results were expressed as picograms of corticosterone per milligram of feather.

### Assessment of blood H:L ratio

The H:L ratio was determined in 5–7 μl of EDTA-treated blood by using the method of Lentfer et al. (2015). In brief, the blood smears were allowed to dry on slides at room temperature and then stained using the rapid panoptic method (MAIM S.L., Barcelona, Spain). After that, samples were examined under an optical microscope with an integrated screen (BioBlue BB.4200-LCD, Euromex Microscopen, Arnhem, Netherlands) to count the blood cell elements, specifically heterophils and lymphocytes, using criteria defined by (Clark et al., 2009). Thus, a total of 100 leucocytes were counted per slide and distinguished as lymphocytes, heterophils, eosinophils, monocytes, or basophils. The number of heterophils was divided by the number of lymphocytes to estimate the ratio between the bulk of the main innate and adaptive immune cells (H:L ratio).

### Statistical analysis

Statistical analyses were conducted with IBM SPSS Statistics software version 29.0.2.0 (IBM Corp., Armonk, NY, USA). Firstly, normality of data was assessed by using the Shapiro–Wilk test and, in case of non-normal distributions, non-parametric tests were applied. The effect of age on biochemical and stress-related parameters was evaluated using the Kruskal–Wallis test. When significant effects were detected, pairwise comparisons between age groups were performed using Dunn’s test with Bonferroni correction. Partial Spearman’s rank correlation analyses were performed to assess associations between metabolic parameters and stress biomarkers while controlling for age, minimizing the influence of physiological changes associated with development and production. Comparisons between the two genetic lines (ML and FL) were performed using the non-parametric Mann–Whitney U test, with Bonferroni correction applied to control for type I error. A probability of *p* < 0.05 was considered statistically significant and all the results are expressed in the manuscript as mean ± SEM.

## Results

### Age-related changes in the metabolic and stress biomarkers

All the metabolism-related parameters were significantly affected by the age of the birds (*p* < 0.01; Fig. 1), although no consistent age-dependent patterns were observed for glucose or NEFA.

**Fig. 1:**
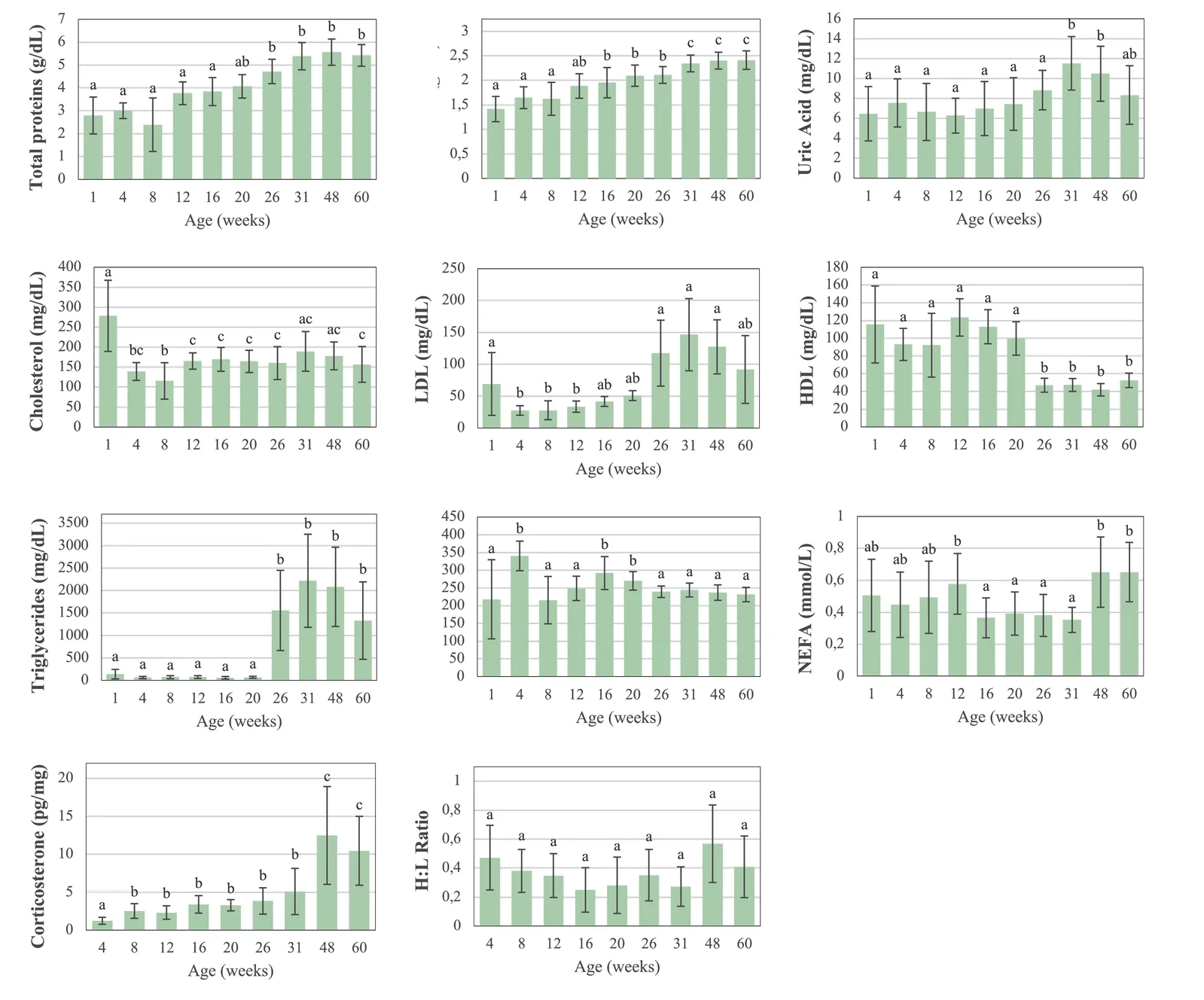
Evolution of metabolic and stress-related parameters in breeder hens from rearing to late lay (4–60 weeks of age). Bars with different letters differ significantly among ages (*p* < 0.05).

Conversely, the concentration of the biomarkers indicative of protein metabolism (total proteins, albumin and uric acid) increased significantly over time of sampling, although uric acid decreased after 31 weeks of age.

On the other hand, biomarkers for lipid metabolism showed distinct patterns over time. Total and LDL cholesterol showed significantly higher values at first week than at subsequent ages (*p* < 0.05) and, after a sharp decline at 8 weeks-old, both parameters steadily increased from Week 8 to 31 to decrease afterwards. In contrast, HDL cholesterol remained at higher concentrations during the first 20 weeks of age, followed by a sharp decrease from Week 26 to 48 and no further significant changes through week 60. Triglycerides followed a pattern similar to that observed for total cholesterol and LDL, remaining at baseline levels up to week 20 and then rising sharply from Weeks 26 to 60.

Assessment of serum stress biomarkers showed different age-related patterns. Corticosterone increased over time, from its minimum at Week 4 to its highest levels at Week 48 (*P* < 0.05). Conversely, the H:L ratio was not significantly affected by age (*P* > 0.05).

### Differences in metabolic and stress biomarkers between pre-laying and post-laying periods

Changes in biochemical parameters and stress biomarkers between the pre-laying and post-laying periods are presented in Fig. 2. All analyzed parameters exhibited significant differences associated with the onset of lay (*p* < 0.01), except for NEFA levels and the H:L ratio, for which no statistically significant differences were detected. Specifically, a significant increase (p < 0.01) was observed in total protein, albumin, uric acid, total cholesterol, LDL-c, triglycerides, and corticosterone levels following the onset of the laying period, whereas HDL-c and glucose concentrations showed a statistically significant decrease (*p* < 0.01).

**Fig. 2:**
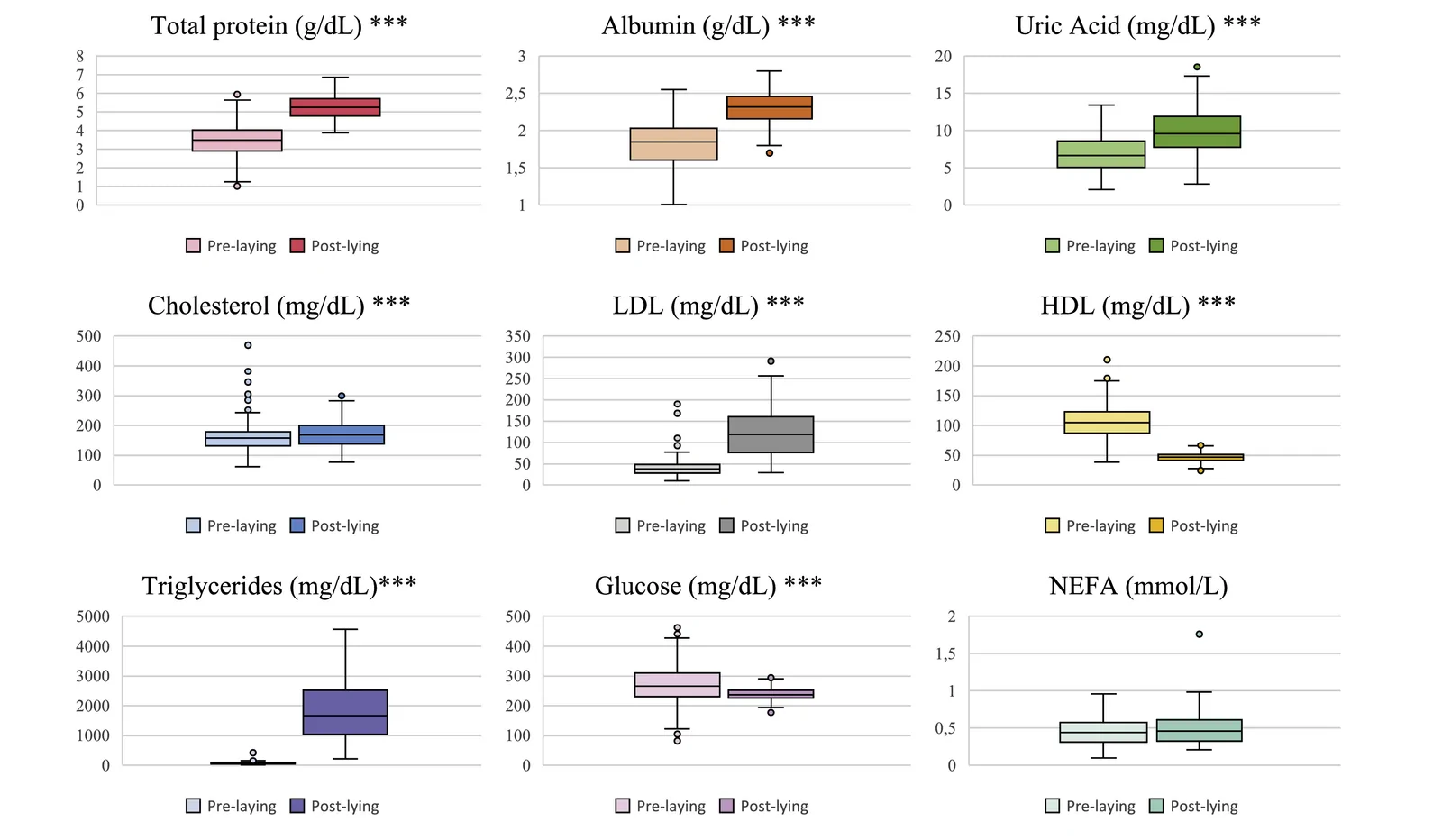

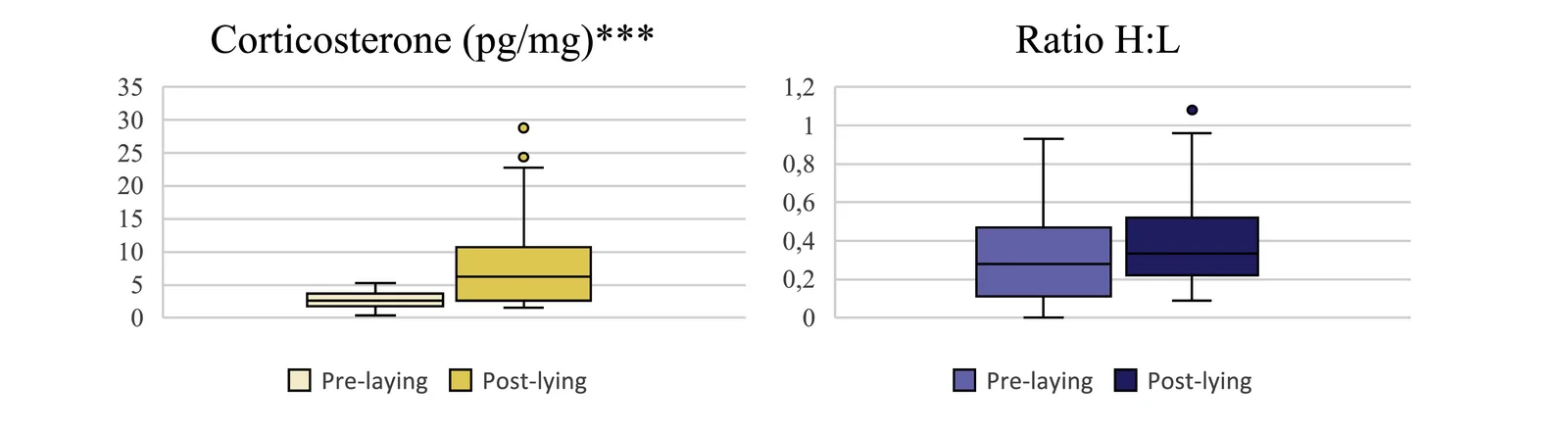
Boxplots depict the median and interquartile range; dots represent individual outlier values. Asterisks indicate *p* < 0.01.

### Reference intervals by age group

Reference intervals (RIs) for each parameter and 90 % confidence intervals (CI) for the lower and upper RI limits are presented in Table 3, grouped according to the different life stages (early rearing, rearing, early laying and laying). Overall, most parameters exhibited a clear age-related shift in their RI.

**Table 3:** Reference intervals for biochemical parameters and stress biomarkers in Cobb broiler breeder hens at different stages of the life cycle.

		Early rearing (4–8 wk)			Rearing (12–20 wk)		
Parameter	SI units	RI	90 %CI for lower limit	90 %CI for upper limit	RI	90 %CI for lower limit	90 %CI for upper limit
Total Protein	g/dL	1.2–5.5	1.0–1.3	4.0–5.9	2.8–5.1	2.5–3.1	4.8–5.3
Albumin	g/dL	1.0–2.1	1.0–1.1	2.0–2.4	1.4–2.5	1.3–1.5	2.4–2.6
Uric Acid	mg/dL	2.4–13.2	2.1–3.3	12.4–13.4	3.0–12.3	2.7–3.6	11.2–12.9
Cholesterol	mg/dL	63.0–233.4	61.9–70.5	175.8–251.9	113.4–213.5	100.1–125.1	207.9–223.2
LDL	mg/dL	10.9–62.8	10.0–11.9	47.9–69.8	20.7–66.2	14.4–23.2	59.1–70.7
HDL	mg/dL	49.0–162.4	45.6–51.3	150.0–174.8	71.2–148.5	65.5–74.1	143.4–180.4
Triglycerides	mg/dL	23.3–148.0	18.1–29.3	120.3–151.0	23.0–127.7	19.9–26.5	114.7–155.2
Glucose	mg/dL	122.7–424.9	101.4–129.9	398.9–447.1	204.1–375.7	194.5–211.8	337.8–410.0
NEFA	mmol/L	0.1–0.9	0.1–0.1	0.9–1.0	0.2–0.9	0.1–0.2	0.8–1.0
Corticosterone	pg/mg	0.1–4.4	0.1 – 0.1	4.3–4.4	0.8–5.2	0.7–0.8	4.8–5.2
H:L Ratio	–	0.1–0.9	0.1–0.2	0.8–0.9	0.0–0.6	0.0–0.1	0.6–0.7

		Early laying (26–31 wk)			Late laying (48–60 wk)		
Parameter	SI units	RI	90 %CI for lower limit	90 %CI for upper limit	RI	90 %CI for lower limit	90 %CI for upper limit
Total Protein	g/dL	4.0–6.6	3.9–4.1	6.2–6.9	4.4–6.5	4.4–4.6	6.4–6.8
Albumin	g/dL	1.8–2.7	1.7–1.9	2.6–2.7	2.0–2.8	2.0–2.1	2.7–2.8
Uric Acid	mg/dL	6.2–16.9	5.7–6.5	15.4–18.6	3.1–15.4	2.8–4.6	14.6–17.4
Cholesterol	mg/dL	98.7–282.2	91.3–116.0	262.3–299.4	84.4–255.6	76.5–102.1	240.8–261.7
LDL	mg/dL	45.2–247.1	41.6–56.5	223.0–291.3	31.9–225.0	29.6–35.8	192.2–227.6
HDL	mg/dL	34.8–65.1	33.9–35.9	61.5–65.5	27.3–67.0	24.5–31.3	58.9–74.6
Triglycerides	mg/dL	254.7–3924.6	218.9–522.5	3730.6–4570.8	318.5–3879.6	282.5–349.3	3292.2–3949.3
Glucose	mg/dL	212.5–292.7	207.9–219.8	269.5–294.9	193.5–282.0	177.2–203.7	267.8–290.6
NEFA	mmol/L	0.2–0.7	0.2–0.2	0.6−0.7	0.2–1.0	0.2–0.4	0.9–1.8
Corticosterone	pg/mg	1.6–10.7	1.6–1.6	9.2–10.7	2.2–24.7	2.2–2.8	20.2–28.8
H:L Ratio	–	0.1 – 0.7	0.1–0.1	0.6–0.7	0.1–1.1	0.1–0.2	0.9–1.1

Biomarkers for protein metabolism exhibited pronounced age-related dynamics. Albumin and total protein presented their lowest RI limits during early rearing, followed by a progressive upward shift throughout rearing and laying, with the highest RI observed during the late laying phase. Uric acid showed relatively lower RI during early rearing and rearing, while wider intervals and higher upper limits were detected during both laying phases.

Biomarkers for lipid metabolism showed the most substantial changes across production stages. Total cholesterol and LDL exhibited relatively lower RI during rearing phases, whereas their upper limits increased markedly after the onset of lay, reaching maximum values during early or late laying. In contrast, HDL displayed higher RI during the rearing period, followed by a clear reduction in both lower and upper limits once the laying phase began. Triglycerides showed the most pronounced increasing of RI, with minimal values observed during rearing and extremely elevated upper limits during the laying phases, reflecting high inter-individual variability linked to egg production.

Glucose concentrations were highest during the early rearing phase. In the subsequent rearing period, values remained elevated but showed a slight decrease. During the early laying phase, the RI narrowed, and a similarly narrower range was observed in the late laying phase. The RI in the case of NEFA remained comparatively stable throughout most of the production cycle, although slightly higher upper limits were observed during late laying, suggesting limited but detectable mobilization of lipid reserves under the management conditions studied.

Finally, feather corticosterone displayed its lowest RI during the early rearing phase, whereas both lower and upper limits increased markedly during the laying period, reaching the highest values in late laying. In contrast, the H:L ratio remained relatively stable across all production phases.

### Correlations between metabolic parameters and stress biomarkers

Correlations among the evaluated stress and metabolic-related biomarkers are presented in Fig. 3.

**Fig. 3:**
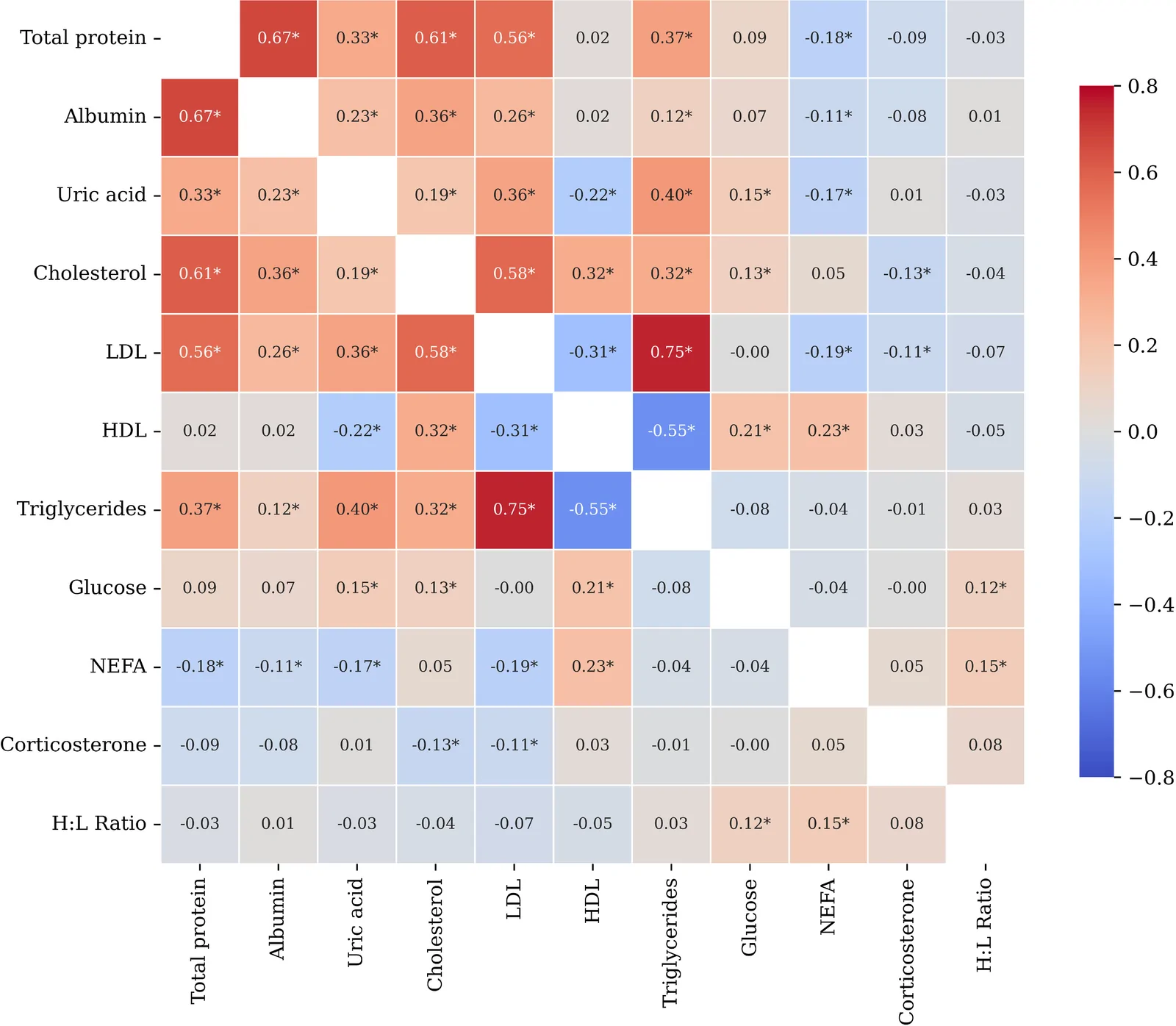
Heatmap of age-adjusted Spearman correlation coefficients among the analyzed variables. Colors indicate the strength and direction of the correlations (blue: negative; red: positive). Asterisks denote statistical significance (*p* < 0.05).

In summary, protein biomarkers (uric acid, albumin, and total proteins) were positively related to all the lipid parameters, excepting HDL-c (i.e.: total cholesterol, LDL-c and triglycerides; *p* < 0.05). Total cholesterol was significantly related to HDL-c, LDL-c and triglycerides (*p* < 0.05). HDL-c showed positive correlations with glucose and NEFA, and negative correlations with LDL-c and uric acid (*p* < 0.05). NEFA showed negative correlations with all protein biomarkers (*p* < 0.05).

On the other hand, corticosterone was negatively correlated with total cholesterol and LDL-c, while the H:L ratio was positively correlated with glucose and NEFA concentrations (*p* < 0.05 for all).

### Effect of the genetic line

There were no significant differences between both genetic lines (ML and FL hens) in any of the metabolic and stress biomarkers studied; excepting uric acid concentrations, which showed higher values in the FL line (Table 4).

**Table 4:** Comparison of biochemical parameters and stress biomarkers between genetic lines (ML vs. FL).

Parameter	Higher line (mean)	Lower line (mean)	p_uncorrected	Difference ( %)	p_corrected (Bonferroni)
Total Protein	FL (4.177)	ML (4.142)	0.526	0.86	1.000
Albumin	ML (2.024)	FL (2.021)	0.645	0.11	1.000
Uric Acid	FL (8.896)	ML (7.423)	0.000	19.84	0.000
Cholesterol	FL (166.879)	ML (162.479)	0.026	2.71	0.290
LDL	FL (81.268)	ML (65.737)	0.022	23.62	0.244
HDL	FL (81.788)	ML (79.983)	0.567	2.26	1.000
Triglycerides	FL (940.112)	ML (649.093)	0.094	44.83	1.000
Glucose	FL (260.015)	ML (250.785)	0.124	3.68	1.000
NEFA	ML (0.473)	FL (0.473)	0.920	0.02	1.000
Corticosterone	ML (4.814)	FL (4.726)	0.154	1.86	1.000
H:L Ratio	ML (0.395)	FL (0.351)	0.277	12.42	1.000

## Discussion

The results of the present study, the first one assessing lifelong changes in metabolic and stress biomarkers in broiler breeder hens to the best of our knowledge, provide useful reference values and intervals from the rearing period to advanced stages of production in a significant number of animals. The availability of such specific reference values will allow the establishment of more accurate standards for monitoring animal health and welfare.

Assessment of the temporal evolution of metabolic parameters in current study showed clear different patterns among short-term energy metabolism (glucose and NEFA) and long-term energy metabolism, body growth and egg production (lipids and proteins).

In the case of both glucose and NEFA, our results indicate the absence of a clear age-related pattern, supporting previous studies (Yang et al., 2013; Ding et al., 2021) and therefore the idea that their dynamics depend more on the short-term metabolic demands characteristic of each productive phase. Thus, as previously described by Schumann et al. (2014) and (Yang et al., 2013) increased energetic demand associated with oviposition decreases glucose concentrations and increases NEFA concentrations. In our present study, significant differences were found when comparing pre- and post-laying phases in the case of glucose (Fig. 2), but changes were modulated in the case of NEFA; likely because hens received a nutritionally balanced diet adjusted to their productive requirements. Maintaining a positive energy balance reduces the need for mobilizing fat reserves, which would explain the absence of significant differences in NEFA concentrations between the pre- and post-laying periods and the relative stability of this parameter throughout the study.

On the other hand, lipid patterns showed significant changes over time. The first sampling, during the first week of life, showed really high concentrations of total and LDL-cholesterol; a finding which is related to the fact that the residual yolk sac constitutes the main source of cholesterol and other lipids for the chick during the immediate post-hatch period, supplying the energetic requirements associated with rapid early growth (van der Wagt et al., 2020). Furthermore, hepatic lipid metabolism is still undergoing maturation during this phase, which is reflected in elevated hepatic and circulating lipid levels; afterwards, total and LDL-cholesterol concentrations gradually decline as the liver reaches functional maturity and dietary intake progressively replaces yolk-derived nutrients (Liu et al., 2020; Han et al., 2025). Finally, cholesterol levels stabilize with the progression of growth, liver maturation and the transition to exogenous feeding, as in previous studies (Yenilmez and Atay, 2023). Conversely, as in previous studies (Gu et al., 2021; Hailemariam et al., 2022; Youssef et al., 2023), LDL-c continues increasing from week 20, a trend associated to the initial physiological preparation for egg production and specifically for favoring yolk formation, reflecting the metabolic demand of vitellogenesis (Máchal, 2000). In fact, LDL-c constitutes the major lipid fraction of the egg yolk, accounting for approximately two-thirds of its total dry matter (Anton, 2007). Thus, the onset of lay induces significant differences between the pre laying and post laying periods for all parameters involved in lipid metabolism, as shown in Fig. 2.

Such significant differences between the pre laying and post laying periods were also found for HDL-c concentrations, although, in contrast, HDL-c declines with age. The marked decline observed from Week 20 onwards has been previously related to the marked increase in circulating estrogen levels occurring during the onset of lay. Estrogens markedly reshape the lipoprotein profile by reducing HDL and increasing very-low-density lipoproteins (VLDL) to meet the lipid demands of vitellogenesis, which highlights the central role of estrogens in regulating hepatic lipid metabolism and lipoprotein synthesis in laying hens (Wang et al., 2013). This estrogen-driven metabolic shift is thus closely associated with the pronounced increase found in the triglycerides concentrations from 20 weeks of age onwards, which is also modulated by estrogen release from the ovary. In fact, estrogens are known to stimulate hepatic lipid metabolism, promoting the synthesis of very low-density lipoproteins (VLDL), which facilitate the transport of triglycerides from the liver to developing oocytes, thereby supporting yolk formation and the progression of reproductive function (Irvine et al., 2018). Afterwards, changes in HDL-c may be related to metabolic changes associated with hepatic aging, so older birds tend to accumulate a higher lipid load in the liver. As a result, a greater proportion of lipids are redirected from circulation toward hepatic and adipose storage, thereby reducing circulating HDL levels (Gu et al., 2021). Higher hepatic lipid retention has been related to increased insulin secretion in adult birds which, concomitantly, also enhances fatty acid synthesis (Honma et al., 2011). This metabolic shift is critical since not only affects the lipid profile but may also influence the overall physiological balance and productive performance of the hen (Kuhla et al., 2011; Moradi et al., 2013; Shini et al., 2019).

The start of the laying period also influences protein metabolism, supporting previous data on albumin concentrations (Harlap et al., 2021); total protein and albumin concentrations increased progressively throughout the production cycle, particularly during oviposition, suggesting an adaptation of hepatic protein synthesis to the increased metabolic demands of egg production, with intensified catabolic processes of amino acids and an increased protein requirement. However, the greatest increase in protein metabolism occurs during the period of highest weight gain and body growth, which in our case takes place from Week 20 onwards, since it is likely related to the high demand for amino acids required for both growth and egg production (Szabó et al., 2005; Filipović et al., 2007). Furthermore, Gyenis et al. (2006) linked the increase in total protein to growth hormone, which stimulates protein synthesis, as well as with the general anabolic processes characteristic of this stage of development, both of which contribute to elevated serum protein levels. The changes in uric acid over time may also be modulated by hormonal shifts associated with the onset of lay, consistent with the metabolic adaptations required for egg production. However, uric acid is also significantly modulated by other factors such as health status, environmental conditions, or feed intake (dos Santos et al., 2010).

Assessment, in the present study, of the interrelationships among the different metabolic parameters shows clear metabolic synergies; mainly, between lipids and protein components. Such findings are consistent with the challenging hepatic metabolism of protein and lipids of laying hens, in which changes in serum proteins, liver enzymes and phospholipids metabolites jointly reflect shifts in lipid deposition and reproductive status (Zhang et al., 2023; Zálešáková et al., 2025; Wang et al., 2026). Overall, these interrelationships reflect coordinated metabolic regulation and indicate that the physiological interpretation of these parameters should be based on their integration rather than on individual values alone (Hrabčáková et al., 2014).

Assessment of stress biomarkers in feathers showed a progressive age-related increase in corticosterone accumulation, a pattern previously described in the literature (Bortolotti et al., 2009). For instance, (Kozak et al., 2019) found corticosterone levels ranging from 3.97 ± 0.17 ng/mL to 4.50 ± 0.19 ng/mL in two strains of 30-week-old hens. Similarly, (Nordquist et al., 2020) observed an upward age-related trend, with approximately 2–3 ng/mL at 16 weeks and 3–4 ng/mL at 28 weeks. However, most published studies provide single time-point measurements or cover relatively limited age intervals, highlighting the need for a more comprehensive and systematic assessment of how feather corticosterone changes throughout the entire life cycle of breeder hens. The present findings contribute to addressing this gap by offering a broader temporal perspective that improves the contextualization and interpretation of this biomarker. Increases in corticosterone concentrations over time were expected because corticosterone is deposited in feathers during their growth, providing an accumulative measure of stress experienced throughout the feather formation period rather than a time-specific snapshot, as occurs with blood sampling (Häffelin et al., 2020). However, in breeder hens, such age-related increase in corticosterone may be also associated with physiological and productive factors. The high energetic and metabolic demands required for sexual maturation and sustained egg production activate the hypothalamic–pituitary–adrenal axis (Hanlon et al., 2020) and increase corticosterone secretion. Thus, the higher concentration observed from Week 31 onwards in our study may be linked to the peak laying period and the maintenance of intensive production, which would explain the differences detected between the pre- and post-laying stages in current study. Conversely, no significant age-related differences were detected on the H:L ratio; the observed ratios fall within the range classified as low or optimal (0.2–0.5) by Campo and Davila (2002) . Similar values have been reported previously; for example, Odihambo Mumma et al. (2006) observed an H:L ratio of 0.34 ± 0.02 in a control group, supporting the stability of this parameter under non-stressful conditions.

The study of the relationship between metabolic and stress biomarkers, an assessment not previously reported to the best of our knowledge, reveals a negative but weak correlation between feather corticosterone and serum total and LDL cholesterol concentrations, supporting the hypothesis that elevated corticosterone may affect hepatic synthesis and metabolism of lipids (Zaytsoff et al., 2019). There was also a positive relationship of the H:L ratio with glucose and NEFA, which is consistent with the role of physiological stress in energy metabolism. Stress response increase plasma glucose (Li et al., 2019; Scanes et al., 2024) because the increase in glucocorticoids occurring under stressful situations promotes the mobilization of hepatic glycogen and glucogenesis, raising therefore plasma glucose concentrations. Concomitantly, stress response also promotes the mobilization of lipids (Nijdam et al., 2005; Zhang et al., 2009), which explains the positive association between H:L ratio and NEFA.

The relationship between age and stress-related biomarkers should therefore be interpreted cautiously, as increasing age in breeder hens is accompanied by important changes in reproductive and metabolic status (Abraham et al., 2025). The onset and establishment of egg production are energetically demanding processes, and corticosterone concentrations may be influenced by the hen's energy balance and physiological state (Leishman et al., 2021). Consequently, the age-related variation observed in feather corticosterone may partly reflect the metabolic and reproductive adjustments associated with sexual maturation and egg production rather than changes in welfare per se. Feather corticosterone provides an integrated measure of glucocorticoid exposure during feather growth (de Jong et al., 2016; Kittelsen et al., 2024) and has been shown to vary across laying stages in turkey hens (Leishman et al., 2021). Similarly, the relatively stable H:L ratio observed in the present study suggests no marked age-related changes in this indicator, although it does not exclude the presence of physiological or metabolic challenges associated with the onset of lay.

Finally, assessment of genetic-related variations in metabolic and stress biomarkers showed, overall, similar profiles but some specific differences. Mainly, FL hens tend to show slightly higher values than ML hens, which may be related to the fact that the FL line has been genetically selected for greater productive efficiency and may therefore present a more active metabolism associated with egg production. In this context, uric acid showed significant differences between the two genetic lines, with higher levels in the FL line. This finding may reflect line-specific differences in reproductive hormonal status, as estrogen is known to stimulate hepatic protein synthesis in laying hens, particularly the production of vitellogenin and very low-density apolipoprotein (Li et al., 2014), a process that increases protein turnover and may indirectly influence plasma uric acid levels. Genetic line had no significant influence on either feather corticosterone or the H:L ratio (Table 4), suggesting that the reference values obtained for these stress biomarkers may be applicable to both the ML and FL lines under similar management conditions.

Initially, we hypothesized that feed restriction would be the main stress-inducing factor, and that the ML line, which has greater growth potential and is therefore subjected to a stricter feeding program, would show higher stress biomarkers than the FL line. Contrary to this expectation, our results showed very similar metabolic and stress profiles between the two lines, with only uric acid differing significantly after Bonferroni correction (Table 4). Instead, the onset of lay emerged as the factor most clearly associated with increases in stress biomarkers (Fig. 2), suggesting that the metabolic and reproductive demands of egg production may represent a more relevant stressor in breeder hens than feed restriction itself under the management conditions studied.

Nevertheless, some limitations should be considered when interpreting the present findings in relation to animal welfare. Although the biomarkers evaluated provide valuable information on physiological stress and metabolic status, they were not assessed in conjunction with other recognized indicators of flock welfare, such as behavioral measures, footpad dermatitis or feather condition (de Jong et al., 2016; Kittelsen et al., 2024) . Therefore, the reference intervals and biomarker values reported should not be considered as standalone measures of welfare status. Future studies combining physiological and metabolic biomarkers with validated behavioral, health and management-based welfare indicators would provide a more comprehensive assessment of the welfare status of breeder hens.

In conclusion, this study provides reference values specific to each age and production stage, offering a comprehensive description of changes in the metabolic and stress biomarkers over time and filling the gap relate to the fact that most published studies provide single time-point measurements or cover relatively limited age intervals. The present findings contribute to addressing this gap by offering a broader temporal perspective that improves the contextualization and interpretation of these biomarkers. The establishment of age-specific reference intervals provides a valuable tool for physiological assessment and welfare monitoring, allowing more accurate detection of potential imbalances. Overall, this study contributes to a better understanding of the physiological mechanisms associated with stress and metabolism in Cobb breeder hens, providing a physiological basis that, combined with other welfare assessment tools, may contribute to improving animal welfare and promoting more sustainable poultry production systems.

## Declaration of AI and AI-assisted technologies in the writing process

During the preparation of this work, the authors used Scopus AI in order to improve the literature search process. The authors also used Claude (Anthropic) in order to assist with language editing and improving the clarity of the writing. After using these tools/services, the authors reviewed and edited the content as needed and takes full responsibility for the content of the publication.

## Author contributions

**Raúl Caballero-Falcó** acquired and analyzed the data, contributing analysis tools for this purpose, and drafted the manuscript. **Antonio González de Bulnes** contributed to the conception and design of the study, participated in data acquisition and analysis, and critically revised the manuscript. **Paula Martínez-Ros** contributed to the conception and design of the study, participated in data acquisition, and critically revised the manuscript. **Juan Carlos Abad** contributed to the conception and design of the study, participated in the acquisition of farm management data, and critically revised the manuscript. **Cristina Guerra** contributed to the conception and design of the study, participated in the acquisition of farm management data, and critically revised the manuscript. **M. Carmen López-Mendoza** contributed to the conception and design of the study, participated in data acquisition and analysis and in the use of dedicated analysis tools, and critically revised the manuscript for important intellectual content. All authors read and approved the final version of the manuscript and agree to be accountable for the integrity of the work.

## Funding

This research was funded by Spain Ministry of Science Innovation and Universities (PLEC2023-010275, MCIN/AEI/10.13039/501100011033).

## Disclosures

Raul Caballero-Falco is supported by a Predoctoral contract from the Universidad Cardenal Herrera-CEU. The other authors declare that they have no known competing financial interests or personal relationships that could have appeared to influence the work reported in this paper.

## Footnotes

Supplementary material associated with this article can be found, in the online version, at doi:10.1016/j.psj.2026.107949.
