# Supplementary material for: Age–related changes in the metabolism and stress biomarkers of breeder hens
Source: Poult Sci. 2026 Oct 14;105(12):107949. doi: 10.1016/j.psj.2026.107949 (PMC13718791; doi:10.1016/j.psj.2026.107949)
Supplement: Supplementary file 2 [file mmc2.pdf]

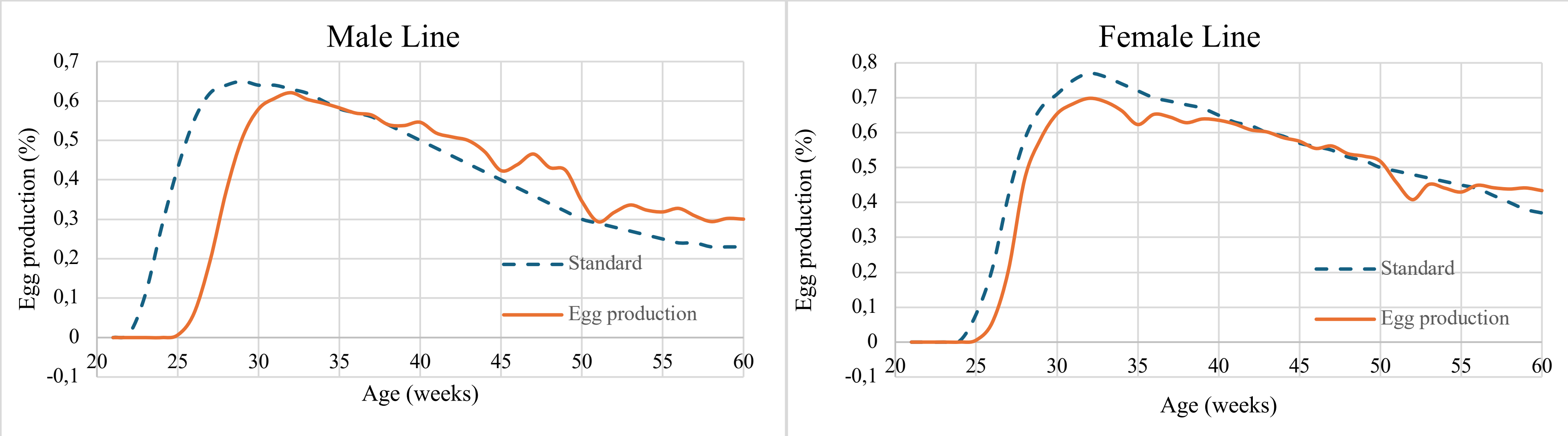

Supplementary Figure S2. Egg production (%) of Female Line and Male Line grandparent hens relative to the Cobb breed standard across age.
